# Dietary variability of western gorillas (*Gorilla gorilla gorilla*)

**DOI:** 10.1371/journal.pone.0271576

**Published:** 2022-08-24

**Authors:** Martha M. Robbins, Sylvia Ortmann, Nicole Seiler

**Affiliations:** 1 Max Planck Institute for Evolutionary Anthropology, Leipzig, Germany; 2 Leibniz Institute for Zoo and Wildlife Research, Berlin, Germany; Universitat Autonoma de Barcelona, SPAIN

## Abstract

Spatial and temporal variability in the availability of food resources will lead to variation in a species’ diet, which can then influence patterns of space use, sociality, and life history characteristics. Despite such potential impacts, little information is available about dietary variability for some species with large geographical ranges. Here we quantify the diet and nutritional content of plants consumed by western lowland gorillas (*Gorilla gorilla gorilla*) in Loango National Park, Gabon over a 2.6 year period and make comparisons with two study sites located 800 km away. The major foods consumed by the Loango gorillas differed greatly from the other two study sites, but gorillas at all three locations spent a similar proportion of feeding time consuming herbaceous vegetation and tree leaves (~ 50%) and fruit (35%). The Loango gorillas spent approximately 10% of feeding time eating nuts, which were not consumed at the other two study sites. Gorillas at those sites spent about 5% of feeding time eating insects, which were not consumed by Loango gorillas. Even though the species composition of the diet differed among the three sites, the nutritional composition of the major food items differed very little, suggesting that western gorillas consume foods of similar nutritional values to meet their dietary needs. This study shows the flexibility of diet of a species with a wide geographic distribution, which has implications for understanding variation in life history characteristics and can be useful for conservation management plans.

## Introduction

The availability of food for a particular animal species varies in both temporal and spatial dimensions: seasonally, inter-annually, and across locations [1–3]. How primates respond to such variability is crucial to their ability to survive and reproduce and relates to many components of a species’ biology including morphology [4], cognitive abilities [5], life history patterns [6], space use [7, 8], and patterns of sociality [9, 10]. Intraspecific variability in food availability and diet also influences species distribution and the density of a species in a given area [11, 12]. Quantifying dietary variability also is useful for developing conservation strategies because it provides information about the constraints and flexibility in a species’ diet and hence the range of suitable habitat [13, 14]. Furthermore, when studying extant great apes, examining dietary variability has implications for understanding the evolution of *Homo* [4].

Intraspecific variation in diet has been observed in all Primate taxa and is largely considered a reflection of temporal and spatial variation in forest composition [14–16]. Among the great apes, intraspecific variability in diet has been examined in habituated orangutans [17], chimpanzees [18–21], and mountain gorillas [22–24], and based on feeding remains from unhabituated western gorillas [25]. In some cases, variability was observed in social groups living in close proximity, whereas in other cases it encompassed large geographic areas.

On a finer scale, understanding how animals meet their nutritional goals or examining the nutritional content of foods in varying environments helps to elucidate their ability to deal with spatial and temporal variability in food availability [3, 26, 27]. While many studies have focused on intraspecific variability in diet composition, less work has been conducted on the variability of nutritional intake [16, 26, 28]. For some primate species, the nutritional content of major dietary items did not differ, even if the species composition and habitats varied [29, 30], whereas in other cases, the nutritional content varied notably for populations living in differing forest types [17, 26]. Primates may strive for nutritional balancing in environments with differing food availability [26, 31]. Overall, more studies are needed of species with large geographic ranges to better understand their dietary and nutritional flexibility and constraints.

The two species of gorillas (*Gorilla gorilla* and *Gorilla beringei*) occupy a wide range of habitats and consume an extensive diversity of plant species [23–25, 32]. Research on gorillas has been heavily biased towards the two small populations of mountain gorillas (*G*. *b*. *beringei*), which live at high altitudes with very low fruit availability and have diets that are primarily herbaceous vegetation and relatively little fruit (<1% and 15% of diet is fruit in the Virunga Volcanoes and Bwindi Impenetrable, respectively [23, 33, 34]). Despite the Bwindi gorillas having slightly lower availability of major food items, more fruit in their diets, and having longer daily travel distances than the Virunga gorillas, the energy intake rates for both populations were similar throughout the year [24]. In Bwindi, when the gorillas were more frugivorous, their intake of protein declined and of carbohydrates was higher than that of Virunga gorillas [28]. Mountain gorillas prioritize consumption of non-protein energy sources (fruit) when available, yet select foods from their habitat that are high in protein, [31].

Due to the challenges of habituating western gorillas to obtain direct observations, the initial studies on the diet of western gorillas were based on indirect measures of diet such as fecal analysis and feeding remains. These studies provide valuable information on species composition of diets, but they give only a coarse analysis of dietary patterns and do not yield important information on food intake and relative importance of particular dietary items [25, 35–38]. Studies of habituated western gorillas revealed that fruit was a major component of the western gorilla diet (about 35%), along with herbaceous vegetation, tree leaves, and insects [35, 39–41]. In addition to the large difference in fruit consumption between gorilla species, western gorillas at one site consume more simple carbohydrates and fiber but less protein than mountain gorillas [39]. These interspecific differences in nutritional intake add to the evidence that aspects of the feeding ecology for the two gorilla species differ in many ways including food availability [36], patterns of space use [42], and diet [25]. Such variability in feeding ecology between species may lead to differences in their social structure and life history patterns, including the later age at maturation for western gorillas and the occurrence of multimale groups in mountain gorillas [32, 43]. However, the studies of western gorilla diet using direct observations of habituated gorillas (as opposed to fecal analysis and/or feeding remains) have come from two field sites located about 60 km from each other [35, 39–41] and may not be representative of western gorillas across their entire range [25].

A preliminary study of western gorillas in Loango National Park, Gabon, based on feeding signs and fecal analysis of non-habituated gorillas found large differences in food availability and diet composition from the two field sites with habituated gorillas (Bai Hokou and Mondika) located 800 km away [36]. The western gorillas in Loango were also observed to consume nuts (the seeds of fruit), which went against predictions given their dentition [44]. Given the large ecological differences among Loango and other western gorilla habitats, questions remain about variability in species composition, proportion of major dietary items in the diet, and the nutritional values of food items for western gorillas across their geographic range. Therefore the specific aims of this paper are:

1. To quantify the dietary species composition of Loango gorillas.
2. Test if frugivory (defined as time spent feeding on fruit) is positively correlated with fruit availability.
3. Determine similarity of dietary species composition at Loango and two other western gorilla research sites (Mondika and Bai Hokou).
4. Compare nutritional composition among major food types of the Loango gorilla diet and compare the nutritional composition of major food items among Loango, Mondika, and Bai Hokou.

## Methods

### Study site and data collection

We studied one habituated group (Atananga Group) of western lowland gorillas in Loango National Park, Gabon, from January 2018 to July 2020. We made observations on 10 individual gorillas, including one adult male (silverback), one blackback male, and eight adult females. To investigate the gorillas’ dietary composition based on feeding time, we recorded the food species and parts consumed by each individual in view using instantaneous scan sampling at ten minute intervals throughout the daily observation period [45]. We categorized food parts into the following food types: fruits (excluding seeds), herbs (leaves, pith and roots from non-woody plants), tree foods (leaves, bark and flowers from trees, leaves from shrubs and lianas; excluding fruit and nuts), nuts (seeds from fruit) and insects (termites and ants). The gorillas consume the seeds when they ingest fruit, but the seeds pass through the gut undigested and are obviously intact in the gorilla feces [36], so ‘fruit’, refers only to the fleshy pulp and skin. Nuts refer to the seeds that are extracted and consumed from particular fruits. For our analysis, we excluded individuals with less than 5 feeding scan entries per day [33], which resulted in the observation of on average three individuals per day (range: 1–7 individuals). Field assistants collected data on the group for a total of 652 days and for an average of 21 days per month (range: 9–29 days). The study group was observed for a total of 5896 hours and for a mean duration of 1965 hours per year (N = 3, range: 1450.7–2384.3). Fruits, flowers from trees, and nuts were available seasonally, whereas herbs and tree leaves were available throughout the year.

Throughout the study, all observers wore surgical face masks when within 20 m distance of the gorillas. From March 2020 onwards, following the outbreak of the SARS Covid-19 pandemic, no visitors were allowed at the project camp and all staff who left the forest for days off or project management reasons quarantined for 14 days prior to residing at the project camp and entering the study area to observe the gorillas. Additionally, the minimum distance maintained between the gorillas and observers was increased from 7 m to 10 m. This study was conducted in compliance with the regulations of the Agence Nationale des Parcs Nationaux and the Centre National de la Recherche Scientifique et Technique of Gabon, and adhered to the American Society of Primatologists’ principles for the ethical treatment of primates.

### Dietary composition based on feeding time

We determined dietary composition by calculating the proportion of daily feeding scan records that included feeding on each food type (i.e. fruits, herbs, tree foods, nuts and insects) for each individual (S1 Table). Additionally, we determined the proportion of daily feeding time from the scan sampling spent on each food item (i.e. combination of food species and part consumed) divided by the total number of feeding records per day and per individual. To determine daily values for the group (N = 652), we averaged daily proportions of feeding on each food type and food item across all individuals. For the monthly values (N = 31 months; S2 Table), we averaged the mean daily values and for the annual values (N = 2 years), we averaged the monthly values. To determine feeding times, we excluded data from 2020 as we did not have information for the whole year. Major food items were classified as those foods that accounted for at least 1% of feeding time for any full year [28].

### Fruit availability index (FAI)

To determine the monthly availability of fruit, we surveyed an average of 192.5 trees per month (range: 60–266) from 27 different species from January 2018 to August 2020. Only tree species whose fruits were consumed by the study group were included. For each month, we monitored on average 8.1 trees per species (range: 1–33) and noted the presence of ripe fruit per tree [36]. Monthly fruit availability was calculated using the following formula:

$$\sum_{\left(k=1\right)}^{n}D_{k}*B_{k}*P_{k}{}_{m}$$

where *D*_*k*_ is the density of fruit species *k* per km^2^ based on vegetation sampling [42], *B*_*k*_ is the mean basal diameter (cm) of species *k* as measured in the phenology, *P*_*km*_ is the percentage of trees of species *k* containing ripe fruits in a given month *m*, and *n* is the total number of tree species [36].

### Nutritional analysis

During this study, we performed nutritional analysis on 33 food items from 26 different species and families, respectively. These food items represented 33% of the diet based on feeding time (N = 61 food items) and 80% of the major foods (N = 25 food items). Between 1 and 7 samples were collected for each species between January 2017 and April 2018 (S3 Table). Plant samples collected were only the parts consumed by the gorillas. We followed the protocol for sample collection as described by [46]. Dried plant samples were analyzed by the Leibniz Institute for Zoo and Wildlife Research in Berlin following the protocol as in [41, 46].

Dried samples were ground with an IKA A 11 Basic mill (IKA®-Werke GmbH & Co. KG, 79219 Staufen, Germany) to a particle size of approximately 1mm. Dry matter content was obtained by drying part of the sample at 105°C overnight. Standard techniques were used for protein (Dumas combustion), fat (petroleum ether extraction, Soxhlett), and energy. We used enzymatic tests, commercial kits from r-biopharm (R-Biopharm AG, 64297 Darmstadt, Germany) for analyses of carbohydrates (sucrose, d-glucose, d-fructose, starch). A lab standard always was run in all nutrient analyses to check for reproducibility and accuracy of the tests. Ash was determined by burning dried samples in a muffle furnace for at least two hours at 550°C, after which only inorganic matter remained and the amount of ash was obtained by difference in weights. Detergent Fiber Analysis, without ash, was used to estimate neutral-detergent fiber (NDF) and acid detergent fiber (ADF), consisting of the three and two main structural carbohydrates of plant cell wall respectively (hemicellulose—only in NDF, cellulose and lignin, which all were measured directly [41]).

### Nutritional quality of diet

To characterize nutritional differences among food types, we compared average macronutrient content of the major gorilla food types. We investigated the following macronutrients: WSC (water soluble carbohydrates), protein, cellulose, hemicelluloses, lignin, NDF (neutral detergent fiber) and ADF (acid detergent fiber). We report nutrient and mean macronutrient values as percentage of dry matter [47]. Additionally, we calculated total non-structural carbohydrates (TNC) by applying the formula: TNC = 100 - %*L* - %*CP* - % *ash* - %*NDF*, where *L* is lipids, *CP* is crude protein, and *NDF* is neutral detergent fiber [24, 47]. Furthermore, we determined the predicted metaboliable energy (PME) per gram of organic matter, applying conversion factors for *CP*, TNC and *L* from nutritional studies of humans [24]. We used the following formula [47]: PME (kcal/g) = [(4 × %*CP*) + (4 × %TNC) + (9 × %*L*) + (1.6 × %*NDF*)]/100.

### Intraspecific variation in diet and nutritional content

To characterize western lowland gorilla dietary diversity, we compared dietary composition, food species and nutritional quality of diet of the Loango gorillas to two other western lowland gorilla sites, Mondika and Bai Hokou. We extracted all data on these sites from published reports [35, 39, 41, 48]. Food species and dietary composition were compared by compiling the percent time spent feeding on important food items at all three sites, defining important food item as those consumed for more than 1% of feeding time [39, 41].

### Statistical analysis

We conducted a Pearson correlation between the availability of fruit per month (monthly FAI value) and the mean proportion of feeding scans that were spent feeding on fruit per month to test whether frugivory was positively correlated with the availability of fruit. Monthly frugivory values were square-root transformed to normalize the distribution of the data. To test whether food types (fruits, herbs, nuts and tree foods) in Loango differed in their mean macronutrient content, we applied Kruskal-Wallis rank sum test [49]. Due to multiple comparisons we adjusted p-values using the Holm method [50]. We also used a Kruskal-Wallis to test for differences in mean macronutrient content of the three food types (fruits, herbs and tree foods) among the three gorilla populations (Loango, Mondika, and Bai Hokou). All test were two-tailed with alpha = 0.05 and performed in R (R Development Core Team, 2011).

## Results

### Dietary composition

Adult members of the study group of western gorillas at Loango National Park consumed 61 different food items (i.e. combination of food species and part consumed), including 35 fruits, 4 herbs, 16 tree foods (leaves, bark and flowers from trees, leaves from shrubs and lianas), 5 nuts and 1 insect (termites from *Cubitermes sp*). Fruits represented the most important part of the diet, accounting for 57% of all food items. We identified 25 food items as major foods as they were consumed for at least 1% of feeding time for any year (N = 2). Those included 12 fruits, 3 herbs, 7 tree foods and 3 nuts (Table 1). Again, fruits constituted the most diverse and important part of the diet, representing 48% of the major food items.

**Table 1:** Important food items, represented as the percentage of feeding time for each food item at Loango National Park, Gabon (this study) and comparisons with two other western lowland gorilla research sites. Important food items are those consumed for more than 1% of feeding time for the duration of each study.

Species	Plant part	Loango	Mondika^1^	Bai Hokou^2^
*Angyocalyx pynaertii*	Fruit	0	0	0.21
*Desplatia dewerei*	Fruit	0	0	1.55
*Dialium bipindense*	Fruit	1.54	0	0
*Dialium pachyphylum*	Fruit	0	0	8.32
*Duboscia macrocarpa*	Fruit	0	2.3	0.85
*Ficus sp*	Fruit	3.74	0	0
*Gilbertiodendron dewevreiiv*	Fruit	0	0.7	1.45
*Haumania danckelmaniana*	Fruit	0	12.0	1.50
*Irvingia gabonensis*	Fruit	1.57	0	0
*Klainedoxa gabonensis*	Fruit	0	3.9	0.44
*Milicia excels*	Fruit	0	1.6	0
*Pancovia laurentii*	Fruit	0	0	1.27
*Pentadesma butyracea*	Fruit	1.83	0	0
*Pseudospondias microcarpa*	Fruit	2.65	0	0
*Pterocarpus soyauxii*	Fruit	2.03	0.7	0
*Sacoglottis gabonensis*	Fruit	2.14	0	0
*Strombosia spustulata*	Fruit	4.16	0	3.26
*Syzygium sp*	Fruit	1.86	0	0
*Tetrapleura tetraptera*	Fruit	0	1.1	0.36
*Tieghemella Africana*	Fruit	1.98	0	0
*Uapaca guineensis*	Fruit	1.98	0	0
*Vitex doniana*	Fruit	5.99	0	0.36
*Afzelia bipindensis*	Leaves	1.25	0	0
*Angyocalyx pynaertii*	Leaves	0	0	2.92
*Anthonotha macrophylla*	Leaves	1.21	0	0
*Baphia laurifolia*	Leaves, bark	8.78	0	0
*Celtis mildbraedii*	Leaves	0	4.9	10.96
*Dialium pachyphylum*	Leaves	0	0	7.21
*Cynometra mannii*	Leaves, flowers	1.82	0	0
*Ficus sp*	Leaves/Bark	1.08	1.4	0
*Milicia excels*	Leaves	4.99	0	0
*Tomadersia sp*.	Leaves	0	0	1.99
*Dalbergia sp*	Leaves, flowers	10.26	0	0
*Whitfieldia elongate*	Leaves	0	2.6	0.16
*Aframomum masuianum*	Pith	4.97	0	0
*Aframomum subsericeum*	Herb	0	3.7	0.05
*Rhynchospora corymbosa*	Pith	6.31	0	0
*Rhynchospora spp*	Stem	0	0	5.04
*Haumania danckelmaniana*	Herb/Stem	9.80	6.2	2.25
*Megaphrynium macrostachyum*	Herb	0	1.3	0
*Palisota ambigua*	Herb/Stem	0	2.6	0.10
*Palisota brachythyrsa*	Herb	0	3.6	0
*Cubitermes sp*	Insect	0	5.6	6.15
*Coula edulis*	Nuts	5.54	0	0
*Calpocalyx heitzii*	Nuts	1.63	0	0
*Dialium pachyphylum*	Seed-dung	0	0	2.84
*Erythrophleum ivorense*	Nuts	6.35	0	0

^1^Lodwick & Salmi 2019;

^2^Masi et al. 2015

Using the proportion of mean annual feeding time spent on each food type (N = 2 years), the study groups’ diet consisted of 31% fruit, 21% herb, 31% tree foods, 10% nuts and 0% insects (Table 2).

**Table 2:** Percentage of feeding time spent consuming different food types of three western lowland gorilla research sites. Values for Loango are an average of the range showed in parentheses for the two full years of this study. Ranges are not given for Bai Hokou and Mondika because the studies were conducted for one year or less.

% Feeding Time	Fruit	Leaves	Herbs	Nuts/Seeds	Insects	Unknown
Loango	31 (28.8–33.5)	31 (28.6–32.8)	21 (18.5–24.3)	10 (9.3–10.8)	0 *	7
Bai Hokou^1^^,^^2^	36	37	10	0	10	7
Mondika^3^	35	24	30	0	6	5

^1^Masi et al., 2015;

^2^Cipoletta et al., 2007;

^3^Lodwick & Salmi, 2020

*3 cases (observed during one scan for all three cases) were reported but two of them were juveniles and hence not included in the analysis and one case was excluded because there were less than 5 feeding scans per day for that adult individual.

### Fruit availability and frugivory per month

**Fig 1:**
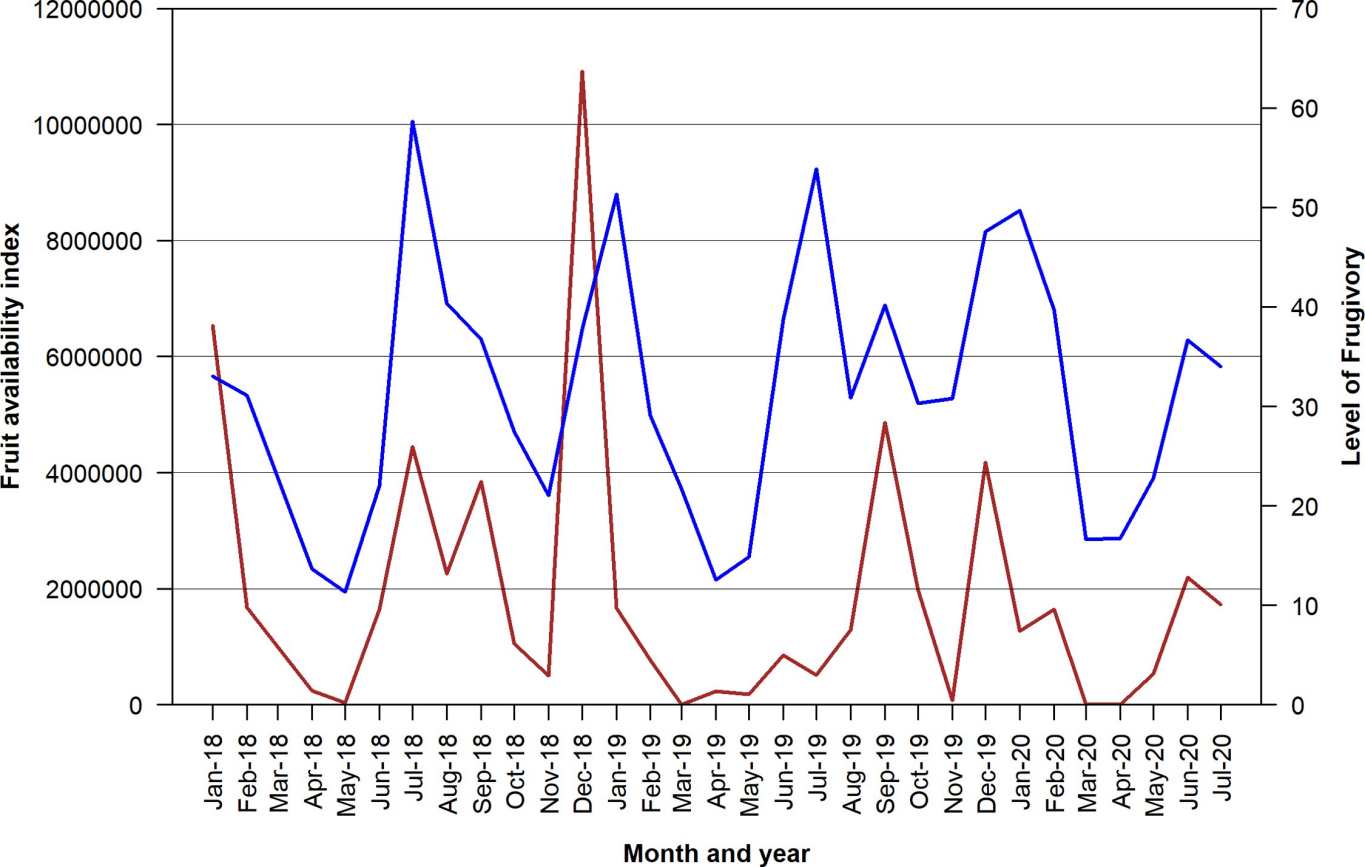
Seasonal changes of the monthly fruit availability (red line) and the monthly level of frugivory (blue line) of one group of western lowland gorillas in Loango National Park from 2018–2020. See Methods for how the fruit availability index was calculated (composite of density of trees, mean basal area of each tree species, and percentage of trees in phenology study with ripe fruit in each month). Level of frugivory is the percent of feeding time spent consuming fruit (see Methods for further explanation).

There were seasonal changes in both the fruit availability per month and the mean proportion of feeding scans that were spent feeding on fruit per month (monthly frugivory). We found a significant positive relationship between the monthly availability of fruit and the monthly frugivory of the group (Pearson correlation coefficient = 0.623, t = 4.209, df = 28, p < 0.001; Fig 1 and S5 Table).

### Nutritional quality of diet in Loango

We found significant differences in the nutritional component of the four different food types for foods consumed by the Loango gorillas (fruits, herbs, nuts and tree foods, Tables 3 and S4). In comparison to tree foods, fruits contained a significantly higher mean concentration of WSC compared to herbs, fruits had a significantly higher mean concentration of TNC. Regarding the mean concentration of protein, both nuts and tree foods showed significantly higher values than fruits. Herbs in Loango tended to have both a higher mean concentration of hemicelluloses than fruits and a higher mean concentration of cellulose compared to nuts. Lastly, nuts showed a significantly higher energetic value compared to herbs and trended to have a higher energetic value compared to tree foods, and fruits showed a trend in having a higher energy value than herbs.

**Table 3:** Macronutritional content of fruits, herbs, nuts and tree foods in the diet of western lowland gorillas in Loango National Park, Gabon.

	Mean % dry matter (except for PME, which is kcal)				K-W-test		Post-hoc-comparison				
Nutrient	Fruit (N = 10)	Herb (N = 3)	Nuts (N = 3)	Tree foods (N = 4)	χ^2^	p	fruits vs herbs	fruits vs nuts	fruits vs tree foods	herbs vs nuts	herbs vs tree foods
WSC	30.8 ± 17.7	4.3±2.6	2.9±1.8	1.8	10.98	**0.012**	Ns	ns	**0.017**	ns	ns
TNC	52.1±12.3	11.2±5.9	34.1±6.8	19.5±6.7	10.36	**0.016**	**0.029**	ns	ns	ns	ns
crude protein	4.9 ± 3.4	11.1±5.1	16.8±9.3	15.7±2.5	11.72	**0.008**	Ns	*0*.*089*	**0.022**	ns	ns
NDF	37.7±20.5	62.7±16.6	27.3±15.0	58.4±8.9	9.31	**0.025**	ns	ns	ns	*0*.*094*	ns
ADF	34.8±20.5	35.1±9.9	10.7±5.8	44.8±12.5	6.76	*0*.*080*	ns	ns	ns	ns	ns
lignin	22.6 ±16.5	7.9±2.7	2.9±1.5	28.4±9.5	7.68	*0*.*053*	ns	ns	ns	ns	ns
hemicellulose	3.0 ±7.3	27.5±5.1	16.6±9.5	13.6±6.0	12.89	**0.005**	*0*.*085*	ns	ns	ns	ns
cellulose	12.2±8.2	27.2±9.9	7.8±5.1	16.5±6.6	7.71	*0*.*053*	Ns	ns	ns	*0*.*094*	ns
PME (kcal)	3.0±1.0	2.0±0.9	4.2±2.4	2.4±1.3	12.1	**0.007**	*0*.*072*	ns	ns	**0.011**	ns

WSC = water soluble carbohydrates, TNC = total nonstructural carbohydrates, ADF = acid detergent fiber, NDF = neutral detergent fiber, PME = predicted metabolizable energy.

Kruskal–Wallis rank sum (K–W) tests were conducted with multiple comparisons adjusted by the Holm method to test for differences among fruits, herbs, nuts, and tree foods. Significant results are in bold, trends are in italic. ns: p≥0.1

### Intraspecific variation in diet and nutritional content

The number of food items that were characterized as major foods based on feeding time was higher in Loango compared to the two other western gorilla populations, possibly because the study period was 2.5 years compared to 1 year at the other sites (Loango: 25, Bai Hokou: 14, Mondika: 15; Table 1) [39, 41]. Based on feeding time, the various food types contributed to the major foods of three different western gorilla populations to differing extents (Table 2). In Loango, fruits represented 48% of the major food items, similar to Bai Hokou with 43%, both being higher compared to Mondika (33%). Leaves were most important in Bai Hokou, representing 29% of the major food items, and Loango (28%) but of less importance for the gorilla population in Mondika (20% of major foods). Herbs constituted 40% of the major foods at Mondika, whereas for Bai Hokou it was 14% and for Loango 12%.

Comparing the time spent feeding on the different food types of the major foods, the level of frugivory was similar across the three sites. The time spent feeding on leaves was highest for Bai Hokou and lowest for Mondika, time spent feeding on herbs was highest for Mondika and lowest for Bai Hokou, with Loango feeding times being in-between (Table 2). The Loango gorillas spent 10% of their feeding time consuming nuts whereas nut feeding was not observed at any of the other two sites. Insect feeding was observed three times in Loango, compared to approximately 6% of time in Mondika and Bai Hokou [35, 39, 41].

Overlap in food species consumed between Loango gorillas and the two populations was very low; only six food items were consumed by the Loango gorillas and the gorillas at the other two sites. In contrast, dietary overlap between the gorillas from Bai Hokou and Mondika was much higher, as they had 12 food items in common. When looking at major food species, overlap of food items among the three sites was even lower. Four major food items were in common between Mondika and Bai Hokou but only two were in common between Loango and Mondiak and between Loango and Bai Hokou.

We found significant differences in the nutritional quality of the major foods among the three western gorilla populations (Table 4). The mean concentration of hemicelluloses in fruits was significantly different among the three populations (Kruskal-Wallis rank sum test: χ^2^ = 8.743, df = 2, p = 0.013) with fruits in Loango (4.76%) having significantly less hemicelluloses than Bai Hokou’s (16.68%; post-hoc test: p = 0.013). Other macronutrients of fruits showed no significant difference among the three populations. Tree foods in Loango contained significantly less protein (15.70%) than in Mondika (21.38%; Kruskal-Wallis rank sum test: χ^2^ = 6.400, df = 2, p = 0.041, post.hoc test: p = 0.042) and significantly more lignin (28.38%) than in Bai Hokou (12.08%; Kruskal-Wallis rank sum test: χ^2^ = 6.218, df = 2, p = 0.045, post.hoc test: p = 0.042). We found no other significant difference in the macronutrient content of tree foods. For herbs, we did not find any significant difference in the nutritional content among the three sites.

**Table 4:** Macronutritional content of fruits, herbs, and tree foods in the diet of three western lowland gorilla populations at Loango, Bai Hokou and Mondika.

	Mean % dry matter			K-W-test		Post-hoc-comparison		
Nutrient	Loango	Bai Hokou	Mondika	χ^2^	p	Loango vs Bai Hokou	Loango vs Mondika	Bai Hokou vs Mondika
Fruit	N = 10	N = 6	N = 5					
WSC	30.8 ±17.1	16.9 ±16.2	19.0±8.2	3.28	0.194			
TNC	52.1±12.3	51.2±23.1	46.2±23.0	0.23	0.892			
Crude protein	4.9±3.4	9.3±3.8	5.4±3.6	4.76	0.093			
NDF	37.7±20.5	35.5±15.5	43.5±20.2	0.55	0.761			
Lignin	22.60±16.5	5.4±5.1	10.7±5.8	5.94	*0*.*051*	0.046	ns	ns
Hemicellulose	3.0±7.3	16.7±8.9	13.7±15.2	8.74	**0.013**	0.013	ns	ns
Cellulose	12.2±8.2	13.4±11.7	19.0±8.0	3.02	0.221			
PME (kcal)	3.0±1.0	3.0±1.3	2.8±0.9	1.22	0.543			
Herbs	N = 3	N = 2	N = 6					
WSC	4.3±2.6	0.3±0.2	3.7±1.1	4.6	0.103			
TNC	11.2±5.9	4.1±2.3	11.1±7.6	1.4	0.494			
Protein	11.1±5.1	22.8±23.3	16.1±4.5	1.3	0.529			
NDF	62.7±5.1	61.2±16.6	54.7±5.7	1.9	0.391			
Lignin	7.9±2.7	7.3±1.0	11.2±4.7	1.7	0.418			
Hemicellulose	27.5±5.1	28.9±6.0	19.1±5.3	4.1	0.130			
Cellulose	27.2±9.9	25.0±11.7	24.4±7.5	0.18	0.913			
PME (kcal)s	2.0±0.9	2.2±1.2	2.2±1.1	0.7	0.717			
Tree Foods	N = 4	N = 4	N = 5					
WSC	1.8±1.0	1.7±2.0	3.1±0.6	2.8	0.242			
TNC	19.5±6.7	28.0±8.8	20.4±7.2	2.4	0.302			
Crude protein	15.7±2.5	21.2±5.2	21.4±3.2	6.4	**0.041**	ns	0.042	ns
NDF	58.4±8.9	44.1±9.9	46.2±9.9	4.08	0.13			
Lignin	28.34±9.5	12.1±5.8	13.2±3.2	6.22	**0.045**	0.042	ns	ns
Hemicellulose	13.6±6.0	18.34±7.5	15.2±6.3	0.69	0.708			
Cellulose	16.5±6.6	13.6±4.8	17.9±4.0	2.2	0.342			
PME (kcal)	2.4±1.3	2.8±0.9	2.7±1.4	4.85	0.089			

WSC = water soluble carbohydrates, TNC = total nonstructural carbohydrates, NDF = neutral detergent fiber, PME = predicted metabolizable energy

Kruskal–Wallis rank sum (K–W) tests were conducted with multiple comparisons adjusted by the Holm method to test for differences among the three western gorilla populations Loango, Bai Hokou and Mondika. Significant results are in bold, trends are in italic. ns: p≥0.1.

## Discussion

This study shows the importance of characterizing diet for species with a wide geographical distribution at multiple locations because it adds to our knowledge that many primates exhibit dietary flexibility across habitats [2, 3]. The diet of western gorillas in Loango National Park, Gabon differed greatly in species composition from two study sites located 800 km away. Such differences are likely to be due primarily to differences in forest composition at the three locations [25, 36]. The total number of major food items (>1% of feeding time) in the gorillas’ diet was greater at Loango than the other two sites, but this was likely due to our study being of longer duration than those at Bai Hokou and Mondika (2.75 years vs approximately one year; [25, 39, 41]). Dietary variability in western gorillas has been shown previously [25], but was based on indirect methodology, so only species composition was compared and not the amount of time spent feeding on particular foods.

Despite the differences in species composition among the three sites, the overall proportion of feeding time consuming fruit was similar (about 35%) among the three study sites (Table 2). Loango gorillas spent nearly 4% of their feeding time consuming figs, whereas figs were not consumed in the other two study sites. Figs are present at Mondika and the gorillas eat the leaves of fig trees [51]. Figs are likely present at Bai Hokou since it is only 50 km away from Mondika, but the density of figs at those sites has not be measured. This is notable because figs are a large component of chimpanzee diet in many locations [20], indicating there may be interspecific competition for figs (and other fruit species) even if the gorillas spend less time consuming them than chimpanzees [36].

Fruit consumption was positively correlated to fruit availability at all three study sites, with feeding time spent consuming fruit on a monthly basis varying from nearly none to as much as 60–70% (Table 1; [35, 41]). Loango differed from the other two sites in that there are two peak fruiting seasons (June–August and December–February) compared to only one (June–September) at Bai Hokou and Mondika [35, 40, 41]. Increased travel distance with increased fruit consumption has been documented at Mondika and Bai Hokou [52, 53]. However, in Loango, the gorillas not only increased travel distance when fruit availability was high, but also were observed to increase their daily travel distance when fruit availability was low but their fruit consumption was still high, suggesting that they were willing to increase travel costs to obtain fruit [42]. Overall, the consistent patterns of frugivory by western gorillas at the three sites confirms that they selectively feed on ripe fruits, similar to other great apes [17, 21, 35].

Tree parts and herbs combined consisted of approximately 50% of the feeding time at all three sites (Table 2). The gorillas at Bai Hokou ate notably less herbs than those at Mondika and Loango. The density of herbaceous vegetation does not appear to explain this difference because Bai Hokou and Mondika had comparable densities of herbaceous vegetation (0.82 and 0.78 stems per m^2^), which were approximately three times higher than that of Loango (0.26 stems per m^2^) [42, 52, 53]. The lack of swamps in the home range of the Bai Hokou gorillas may explain the difference because the Loango and Mondika gorillas spent significant time feeding in swamps containing high densities of herbs [42, 53]. The Bai Hokou gorillas may have compensated for their lower time consuming herbs compared to the other two sites by spending more time eating tree leaves (Table 2).

The Loango gorillas only rarely consumed termites, whereas termites consisted of about 6% of the diet for the gorillas at the other two study sites [35, 39, 54]. Large termite (*Cubitermes sp*.) mounds do not occur in Loango, which likely explains the lack of termites in the Loango gorilla diet. Termites are high in protein, so the Loango gorillas may have less protein intake than at the other sites. In constrast, the Loango gorillas spend about 10% of their feeding time consuming nuts (and as much as 41% in one month), which has not been observed at other field sites. Termite feeding at Bai Hokou and Mondika occurs during all months, whereas the nut eating in Loango was seasonal (December–February).

The nutritional values of different plant parts at Loango differed in a similar manner as was observed at Mondika (Table 3; [39]), although fewer differences were observed possibly due to a smaller sample size of plants at Loango. Fruits had less protein than leaves and herbs at Mondika, whereas in Loango fruit had less protein than tree foods and nuts, but not herbs. Fruits had more total non-structural carbohydrates than leaves and herbs at Mondika, but in Loango fruits had more TNC than herbs but not more than tree foods or nuts. The results from Loango support the concept that the gorillas are selecting fruit for non-protein energy content [39]. Furthermore the lack of difference in nutritional content of herbs and leaves (tree foods) at Loango provides additional evidence that western gorillas may use them interchangeably as protein sources [39].

Very few differences in the nutritional values of major foods among the three sites were observed (Table 4). Tree foods in Loango had lower amounts of protein and greater amounts of lignin than leaves at the other two sites. The only difference in nutritional content of fruit among the three sites was that fruit at Loango had lower values of hemicellulose than fruit at Bai Hokou. No differences were observed in the nutritional content of herbs at the three sites. Thus, although the plants consumed at the three sites varied greatly, the gorillas appear to be selecting food items with similar nutritional content, namely fruits containing high amounts of carbohydrates and herbs and leaves high in protein [39, 40]. Therefore forest composition on the level of nutritional content may be more influential in determining gorilla presence/abundance as actual plant species composition.

The similarity of time spent foraging on the major food categories and nutritional content of major foods among the three sites suggests that western gorillas may work to obtain similar energy and dietary intake by seeking to optimize energy and protein intake [26, 27, 31]. However, we cannot say if they have similar energy balance because we could not calculate overall nutritional intake without food intake rates for Loango and direct comparisons of energy expenditure are not possible. The seemingly longer daily travel distances of Loango gorillas and the lower density of gorillas compared to gorillas at the other two sites may be due to the lower availability of terrestrial herbaceous vegetation [42, 55], but it may also be influenced by the availability of fruit and tree leaves.

Food availability is expected to influence the life history characteristics and density of a species [12]. Greater energy intake and less energy expenditure resulting from greater food availability should result in earlier age of first reproduction, shorter interbirth intervals, and higher lifetime reproductive success, if mortality rates are held constant (e.g. [56, 57]). Variation in some life history characteristics between mountain gorillas and western gorillas have been documented, namely longer interbirth intervals in western gorillas [43, 58, 59]. However, variation in life history characteristics among western gorillas has not yet been documented. Depending on rates of mortality, differences in fertility could influence population growth rates and population density [59].

The density of western gorillas varies greatly across Central Africa, which likely is due to a combination of variation in ecological conditions (including interspecific competition) and/or anthropogenic disturbances [60]. The density of terrestrial herbaceous vegetation has been considered to be a key influence on the density of western gorillas, based on their consumption of species of the Marantaceae and Zingiberaceae families in swamps and on *terra firma* ground [38, 61–64]. The gorillas in Loango are found more in swamp and secondary forest than in coastal and primary forest, further suggesting their preference for habitat that contains a high abundance of herbaceous vegetation [65]. Nonetheless, considering that herbaceous vegetation in the three studies compared here makes up as little as 10% and as much as 30% of feeding time for western gorillas, the relationship between herbaceous vegetation and gorilla density warrants further investigation, namely through the study of diet in areas with very high densities of gorillas and/or terrestrial herbaceous vegetation. Though based on only three studies, it appears that western gorillas may compensate for low amounts of terrestrial herbaceous vegetation in their diet by eating tree leaves, which differ little nutritionally from herbaceous vegetation [39]. The density of important tree food species may also play a role in determining gorilla density [25]. To date, no comparisons of abundance of tree species that contribute to gorillas’ key food items, tree leaves and fruit, have been conducted.

Overall, while this study adds to our knowledge of the flexibility of diet for a species with a wide geographic distribution, more information is needed to better understand the relationship between food availability and dietary variability in western gorillas. It is clear that caution should be taken when attempting to extrapolate from one area to another how the role of food availability influences other parameters such as population density. Furthermore, studies examining the relationship between ecological conditions and gorilla densities will be confounded by levels of anthropogenic impacts. Information on dietary variability is valuable not only for theoretical topics such as life history variation and the evolution of extinct Hominin taxon [4], but also for the development of conservation management plans because it expands our understanding of habitat suitability [66]. Extrapolating dietary patterns from one location to a broader geographic area may lead to incorrect conclusions concerning habitat suitability for extinct species and endangered species.

## Supporting information

S1 Table Observational data (instantaneous scans) per individual gorilla to estimate diet. (XLSX)

S2 Table Proportion of feeding time spent feeding on each species per month. (XLSX)

S3 Table Date, time, and location of plant sample collection used for nutritional analysis. (XLSX)

S4 Table Nutritional content of major foods consumed by the Loango gorillas. (XLSX)

S5 Table Monthly values of percentage of diet that was fruit and the fruit availability index (FAI). See Methods section for how FAI was calculated.
(XLSX)
